# Integration of an [FeFe]-hydrogenase into the anaerobic metabolism of *Escherichia coli*

**DOI:** 10.1016/j.btre.2015.10.002

**Published:** 2015-10-19

**Authors:** Ciarán L. Kelly, Constanze Pinske, Bonnie J. Murphy, Alison Parkin, Fraser Armstrong, Tracy Palmer, Frank Sargent

**Affiliations:** a School of Life Sciences, University of Dundee, Dundee, Scotland DD1 5EH, UK; b Department of Chemistry, Inorganic Chemistry Laboratory, University of Oxford, Oxford OX1 3QR, UK; c Department of Chemistry, University of York, Heslington, York YO10 5DD, UK

**Keywords:** Bacterial hydrogen metabolism, Fermentation, Protein engineering, Molecular genetics, [FeFe]-hydrogenase, Electron-bifurcation

## Abstract

Biohydrogen is a potentially useful product of microbial energy metabolism. One approach to engineering biohydrogen production in bacteria is the production of non-native hydrogenase activity in a host cell, for example *Escherichia coli*. In some microbes, hydrogenase enzymes are linked directly to central metabolism via diaphorase enzymes that utilise NAD^+^/NADH cofactors. In this work, it was hypothesised that heterologous production of an NAD^+^/NADH-linked hydrogenase could connect hydrogen production in an *E. coli* host directly to its central metabolism. To test this, a synthetic operon was designed and characterised encoding an apparently NADH-dependent, hydrogen-evolving [FeFe]-hydrogenase from *Caldanaerobacter subterranus.* The synthetic operon was stably integrated into the *E. coli* chromosome and shown to produce an active hydrogenase, however no H_2_ production was observed. Subsequently, it was found that heterologous co-production of a pyruvate::ferredoxin oxidoreductase and ferredoxin from *Thermotoga maritima* was found to be essential to drive H_2_ production by this system. This work provides genetic evidence that the *Ca*.*subterranus* [FeFe]-hydrogenase could be operating *in vivo* as an electron-confurcating enzyme.

## Highlights

• Engineering microbial biohydrogen production may have biotechnological applications.
• A synthetic operon encoding an NADH-linked [FeFe]-hydrogenase was designed.
• The enzyme was heterologously produced, activated and characterised.
• The addition of ferredoxin and pyruvate oxidoreductase was necessary for *in vivo* activity.

## Graphical abstract

**Figure:**
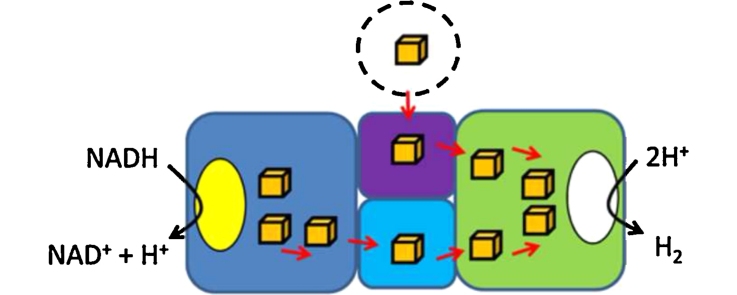

## 1 Introduction

Biohydrogen (Bio-H_2_), which is hydrogen produced biologically from sustainable sources, is a possible future source of biofuel or industrial chemical feedstock [38]. While hydrogen metabolism is rare in higher eukaryotes, many microorganisms naturally produce H_2_ in order to dispose of excess reducing equivalents under some growth conditions. Various anaerobic bacteria are capable of H_2_ production during fermentation. Typical Bio-H_2_ yields from fermentation are 2 mol of H_2_ per mol of glucose with typically 1.3 mol H_2_ per mol glucose being achieved [14], [37] and it has been suggested that in order for Bio-H_2_ to become commercially viable yields must increase to >6 mol H_2_ per mol of glucose [7]. Heterologous production of hydrogenases, the enzymes responsible for the majority of biological H_2_ production (and oxidation), from other H_2_-producing organisms is an attractive strategy to improve Bio-H_2_ yields since non-native hydrogenases that have alternative catalytic properties, cellular localisations or substrate specificities may offer advantages.

The two main classes of bacterial hydrogenases are named [FeFe] and [NiFe] according to the metals in their respective active sites. In both cases, the Fe ions carry additional CO and CN^−^ ligands [18]. Also in both cases, additional accessory proteins are required for the biosynthesis of the metal cofactors with their non-proteinaceous ligands [8], [12]. [FeFe]-hydrogenases typically have H_2_-production activities that are 10–100 times greater than [NiFe]-hydrogenases and are therefore attractive candidates for projects aimed at studying H_2_ production [1], [19]. For biosynthesis of the active site of an [FeFe]-hydrogenase at least three accessory proteins (HydE, HydF and HydG) are required [8]. Thus, for producing [FeFe]-hydrogenases in a non-native host such as *Escherichia coli*, heterologous co-production of these accessory proteins is essential for recovery of active enzyme [2], [30], [42].

*E. coli* can perform a mixed-acid fermentation and under these growth conditions, where respiratory electron acceptors are limited, the cell faces the challenge of re-cycling NAD^+^ from the NADH generated by glycolysis. *E. coli* normally tackles this problem by producing an alcohol dehydrogenase (AdhE), which combines two activities into a single protein: acetyl CoA-dependent aldehyde dehydrogenase and alcohol dehydrogenase [22]. Both reactions utilise NADH as reductant and the resultant ethanol is excreted from the cell. An *adhE* mutant is therefore severely compromised in its growth under strict fermentative conditions [13], [15], [24], [35]. In other biological systems it is possible to link cofactor cycling directly to H_2_ metabolism. *Ralstonia eutropha* (re-named *Cupriavidus necator*), for example, produces a soluble complex between a diaphorase and a [NiFe]-hydrogenase that allows NADH production in a H_2_-dependent manner [10]. *Caldanaerobacter subterranus* subsp*. tengcongensis* (formerly *Thermoanaerobacter tengcongensis*) is a thermophilic Gram-negative bacterium [58] that produces a cytoplasmic [FeFe]-hydrogenase originally reported to produce H_2_ with NADH as sole electron donor [52]. The prospect of an [FeFe]-hydrogenase biased towards H_2_ production, linked directly to NADH oxidation, mades the *Ca. subterranus* enzyme very attractive for potential Bio-H_2_ applications. The *Ca. subterranus* enzyme comprises a complex of four subunits, HydA-D [52]. The HydA subunit is an [FeFe]-hydrogenase predicted to contain an active site ‘H’-cluster as well as four other Fe-S clusters; the HydB subunit is predicted to be a flavin-containing diaphorase subunit with three additional Fe-S clusters; and HydC and HydD are small electron-transferring proteins each predicted to harbour a single Fe-S cluster. All four proteins have been co-purified in a single complex [52].

In recent years, electron-bifurcating hydrogenases, which direct electrons from H_2_ oxidation to two different acceptors, and electron-confurcating hydrogenases, which simultaneously receive electrons from two different sources [49], have been described in various biological systems [9], [50], [51], [56]. In the example of an electron-confurcating enzyme an [FeFe]-hydrogenase receives electrons from NADH and reduced ferredoxin, which together drive H_2_ production and recycling of NAD^+^ [51]. The source of reduced ferredoxin varies between biological systems, but could be linked to pyruvate::ferredoxin oxidoreductase (POR) [56]. Despite the initial report that the *Ca. subterranus* [FeFe]-hydrogenase receives electrons only from NADH [52], this enzyme shares considerable overall sequence identity (43–56%) with the subunits of the [FeFe]-hydrogenase from *Thermotoga maritima* that has been characterised as an electron-bifurcating enzyme [51].

In this work, the overall aim was to engineer an NADH-dependent [FeFe]-hydrogenase into *E. coli* central energy metabolism. Although from a thermophilic bacterium, the *Ca. subterranus* NADH-dependent [FeFe]-hydrogenase was a very attractive candidate given its probable bias towards H_2_ production and its diaphorase activity linked directly to its hydrogenase activity [52]. To this end, a synthetic version of the *Ca. subterranus* NADH-dependent [FeFe]-hydrogenase was designed, constructed and activated. An *E. coli* strain was then constructed where the synthetic operon encoding the *Ca. subterranus* enzyme replaced *adhE* at its native chromosomal locus. This *E. coli* engineered strain was tested for H_2_ production, but no gas production was evident. However, co-production of *Th. maritima* ferredoxin and POR in the engineered strain was found to able to induce low but detectable amounts of hydrogen production. Further genetic experiments led to the conclusion that the *Ca. subterranus* NADH-dependent [FeFe]-hydrogenase could likely operate *in vivo* as an electron-confurcating enzyme. This work provides first proof-of-concept evidence that an active NADH-linked [FeFe]-hydrogenase can be produced in *E. coli*, and that this enzyme has the potential to be further engineered for bioenergy applications.

## 2 Experimental procedures

### 2.1 Bacterial strains and growth conditions

Strains constructed in this work are listed in Table 1. The FTD147h3 strain, which carries a synthetic operon encoding the *Ca. subterranus* NADH-dependent [FeFe]-hydrogenase in place of *adhE*, was constructed as follows: a DNA fragment of approximately 500 bp upstream of the *adhE* gene, including all regulatory elements, was amplified by PCR and cloned into pBluescript (Amp^R^) as a KpnI/EcoRI fragment. Next, a 500 bp fragment covering the *adhE* stop codon and downstream sequence was amplified and cloned as a HindIII/SalI fragment in the same vector, thus resulting in a pBluescript-encoded Δ*adhE* allele. Next, this plasmid was digested with EcoRI/HindIII and the synthetic *hydC-tte0891-hydD-hydB-hydA* operon inserted. The new Δ*adhE*::(*hydC-tte0891-hydD-hydB-hydA*) allele was then transferred to pMAK705 and on to the chromosome of FTD147 as described [25]. The strain Teatotal1 was constructed by moving the unmodified Δ*adhE* allele from pBluescript onto pMAK705 and from there onto the chromosome of FTD147. The FTD147h3 strain was further modified by the addition of an Δ*iscR* allele to yield strain CLK001. Strains CPD152E and CPD159F were constructed by introducing the Keio Collection Δ*pflA*::*kan* allele [4] or a *pflA*::Tn*5* transposon insertion [23] into strains CLK001 and FTD147h3, respectively by P1_*kc*_ mediated transduction [48].

### 2.2 Plasmid construction

A list of the key plasmids studied in this work is provided in Table 1. For the construction of the synthetic operon encoding the *Ca. subterranus* NADH-dependent [FeFe]-hydrogenase, the primary amino acid sequences of the products of *hydC*, *tte0891, hydD, hydB,* and *hydA* were back-translated into DNA sequence, which was then codon optimised using the OPTIMZER software [43] with codon adaptation indices of between 0.7–0.8. Appropriate restriction enzyme sites were chosen and inserted and a strong ribosome binding site and linker sequence analysed using RBS CALCULATOR [46] and inserted before each gene. This final sequence was then synthesized as a service by Biomatik Corp (USA). The synthetic operon was sub-cloned into the *E. coli* production vector pUNI-PROM (Amp^R^; P_T7_; P_*tat*_) [28] as a BamHI and SalI fragment resulting in the plasmid pUNI-Tte-Hyd. To delete individual hydrogenase genes, thus allowing facile identification of produced gene products, pUNI-Tte-Hyd was digested with: *Spe*I (pUNI-Tte-HydΔC); *Bgl*II (pUNI-Tte-HydΔ0891); *Sac*I (pUNI-Tte-HydΔD); *SphI* (pUNI-Tte-HydΔB); *Xho*I (pUNI-Tte-HydΔA); and *Sph*I and *Xho*I (pUNI-Tte-HydΔAB).

To fuse an N-terminal hexa-Histidine tag to HydC, the *hydC* gene was amplified using the oligonucleotides TtehydCNTermHisfor (GCGCACTAGTAGGAGGAAAAAAAAATGCACCATCACCATCACCATCAAGGTATGAAAGAGGCG) and TtehydCNTermHisrev (GCGCACTAGTTTATTCGAACTTTTTCAGGATTTCG) and subsequently digested with *Spe*I and cloned into *Spe*I-digested pUNI-Tte-Hyd, resulting in pUNI-Tte-Hyd_hisC_.

To construct plasmids that would encode accessory genes required for [FeFe]-hydrogenase biosynthesis, the four gene operon containing *SO_3923* (*hydG*); *SO_3924* (*hydX*); *SO_3925* (*hydE*) and *SO_3926* (*hydF*) was amplified by PCR from *Shewanella oneidensis* genomic DNA, digested with *Bgl*II/*Hind*III and cloned into *Bam*HI/*Hind*III-digested pUNI-PROM and pSU-PROM (Kan^R^; P_*tat*_) to give the plasmids pUNI-Sh-EFG and pSU-Sh-EFG. To delete sections of the *Sh. oneidensis hydGXEF* operon to allow identification of produceed genes, pUNI-Sh-EFG was digested with: *Sac*I (pUNI-Sh-EFG_SacI_); *Cla*I (pUNI-Sh-EFG_ClaI_); *Xho*I (pUNI-Sh-EFG_XhoI_); and *Nco*I (pUNI-Sh-EFG_NcoI_).

In order to separate each half of the *Sh. oneidensis hydGXEF* operon, *hydGX* and *hydEF* were amplified using the oligonucleotides DRGXfor (GCGCGAATTCAGGAGGAAAAAAAAATGAGCACACACGAGC), DuetShGtoXrev (CGCGAAGCTTTCATCTGTTAAACCC) and DREFfor (GCGCAGATCTAGGAGGAAAAAAAAATGATCACTCGCCCTAGC), newDuetShEtoFrev (CGCGGACGTCCTATTGCTGAGGATTGCGG), respectively, before the *hydGX* EcoRI/HindII and *hydEF* BglII/AatII fragments were subsequently cloned into pACYCDuet1 resulting in pDuet-Sh-GX-EF. The *hydGX*-*hydEF* fragment was then subcloned into the production vector pSU23 and an *Eco*RI/*Hind*III, fragment, resulting in pSU23-Sh-GX-EF. Finally, in order to insert the constitutive promoter of the *E. coli tat* operon (P_*tat*_) upstream of *hydG*, the *tat* promoter was amplified using the following oligonucleotides: tatfor (GCGCGAATTCTGTCGGTTGGCGCAAAACACGC) and tatrev (GCGCGAATTCCTGTGGTAGATGATGATTAAACAAAGC), which was then digested with EcoRI and cloned into pSU23-Sh-GX-EF, resulting in pSUtat-Sh-GX-EF.

The *Thermotoga maritima* gene *tm0927* (encoding a ferredoxin), and the operon *tm0015-tm0018* (encoding the *γ*, *δ*, *α* and *β* subunits of a pyruvate-ferredoxin oxidoreductase), were amplified by PCR using *Th. maritima* genomic DNA as template (a gift from the group of Michael Adams, University of Georgia) and cloned into pUNI-PROM, yielding pUNI-Tm-Fd6 and pUNI-Tm-POR, respectively. The pUNI-Tm-POR plasmid was further modified by the addition of the tm0927 gene, yielding a plasmid that would encode all five genes from *Th. maritima* (pUNI-Tm-POR-Fd).

All constructs made during this study were sequenced on both strands to ensure that no undesired mistakes had been introduced during the amplification procedure.

### 2.3 Protein methods

For gene product synthesis tests *E. coli* strain K38/pGP1-2 [53] was transformed with the required plasmid. Synthesis of plasmid-encoded gene products was induced by heat shock and followed by labelling with ^35^S-Methionine as described previously [53]. Samples were separated by SDS-PAGE (12% w/v acrylamide) after which gels were fixed in 5% (v/v) acetic acid, 10% (v/v) methanol, dried, and proteins visualised by autoradiography.

Isolation of the synthetic histidine-tagged hydrogenase complex was carried out by Immobilised Metal Affinity Chromatography (IMAC). 5 L of LB supplemented with 0.4% (w/v) glucose, 2 mM cysteine and 2 mM ferric ammonium citrate and antibiotics was inoculated and grown anaerobically at 37 °C for 16 h. All buffers used throughout purification were saturated with N_2_ to remove O_2_ and cell pellets, cell-containing buffers, or crude extracts were flushed with argon to protect from O_2_. Cells were harvested by centrifugation and pellets resuspended in either 50 ml of B-PER^®^ solution (Thermo Scientific), which is a detergent-based cell lysis cocktail, or 50 ml of 50 mM Tris.HCl pH 7.5, 1 mM DTT, 2 mM flavin mononucleotide, 150 mM NaCl and 25 mM imidazole. B-PER lysis was achieved by the addition of lysozyme and DNAse I followed by agitation at room temperature for 1 h. Sonication was also preceded by the addition of lysozyme and DNAse I and the following conditions were used to lyse the cells using a 102-C sonication horn (Branson) and Digital 450 Digital Sonifier (Branson): 20% amplitude; 5 s pulse on/off; and lysis duration of 20 min (40 min total). Following either method of lysis, unbroken cells were removed by centrifugation and resultant crude extracts were applied to 5 ml HisTrap HP affinity columns (GE Healthcare) at a flow rate of 0.5 ml min^−1^. The columns had been previously equilibrated with N_2_-saturated Ni-purification buffer A (50 mM Tris.HCl pH 7.5, 1 mM DTT, 150 mM NaCl and 25 mM imidazole). A linear gradient of 0–100% buffer B (50 mM Tris.HCl pH 7.5, 1 mM DTT, 150 mM NaCl and 1 M imidazole) was then applied to the column to elute bound proteins.

Size exclusion chromatography coupled with multi-angle laser light scattering (SEC-MALLS) was performed using a Dionex Ultimate 3000HPLC system, a MAbPac SEC–1 (Dionex) column, an inline miniDAWN TREOS (Wyatt) multi-angle laser light scattering detector and a T-rEX (Optilab) refractive-index detector. After equilibration with 1.5 column volumes of SEC buffer (50 mM Tris.HCl pH 7.5, 150 mM NaCl), 500 μl of protein was applied to the column at a flow rate of 0.5 ml min^−1^. ASTRA v6.0.0.108 (Wyatt) software was used to determine molecular mass, polydispersity and radius of the enzyme complex.

SDS–PAGE was performed as described [33], and Western blotting was according to [54]. Monoclonal penta-His antibody was obtained from Qiagen. Protein identification was performed by tryptic peptide mass fingerprinting (Fingerprints Proteomics Service, University of Dundee).

### 2.4 Enzymatic assays

H_2_-dependent reduction of benzyl viologen (BV) was assayed by monitoring the reduction of BV at A_600_ as described [39]. H_2_-production using methyl viologen (MV) as electron donor was monitored in a Clark-type electrode modified to measure H_2_. Typically, 2 ml of anaerobic buffer (100 mM sodium phosphate pH 6.0 or 6.8) was added to the reaction chamber together with 12.5 mM MV and 650 μM sodium dithionite and allowed to equilibrate. The reaction was initiated by the addition of enzyme or cell extract and recorded at 37 °C.

### 2.5 Gas and metabolite quantification

Strains for H_2_ and metabolite analysis were grown anaerobically in either a supplemented M9 medium containing M9 salts [48], 2 mM MgSO_4_, 0.1 mM CaCl_2_, 0.2% (w/v) casamino acids, 3 μM thiamine hydrochloride, trace element solution SL-A [26], 0.8% (w/v) glucose or in TGYEP, pH 6.5 containing 0.8% (w/v) glucose [6].

In order to determine hydrogen content in the headspace of anaerobically grown cultures using gas chromatography, Hungate tubes were initially filled with 5 ml of medium and the headspace (approx. 10 ml) was flushed with nitrogen. After 17 h of growth 500 μl aliquots of the gas in the headspace was analysed on a Shimadzu GC-2014 gas chromatograph. Pure nitrogen was used as the carrier gas with a flow of 25 ml min^−1^, and the amount of hydrogen in the headspace was calculated based on a standard curve.

For organic acid analysis, the cell supernatants were passed through a 0.22 μM sterile filter and 10 μl applied to an Aminex HPX-87H (300 × 7.8 mm) ion exchange column. The flow was 0.5 ml min^−1^ at 50 °C and 5 mM sulfuric acid used as mobile phase on an Ultimate 3000 LC system. Organic acid retention peaks were recorded using Chromeleon 6.8 software (Dionex) and quantified by comparison with absorption of known amounts of standard of the organic acids.

## 3 Results

### 3.1 Design, construction and characterization of a synthetic [FeFe]-hydrogenase operon

**Fig. 1:**
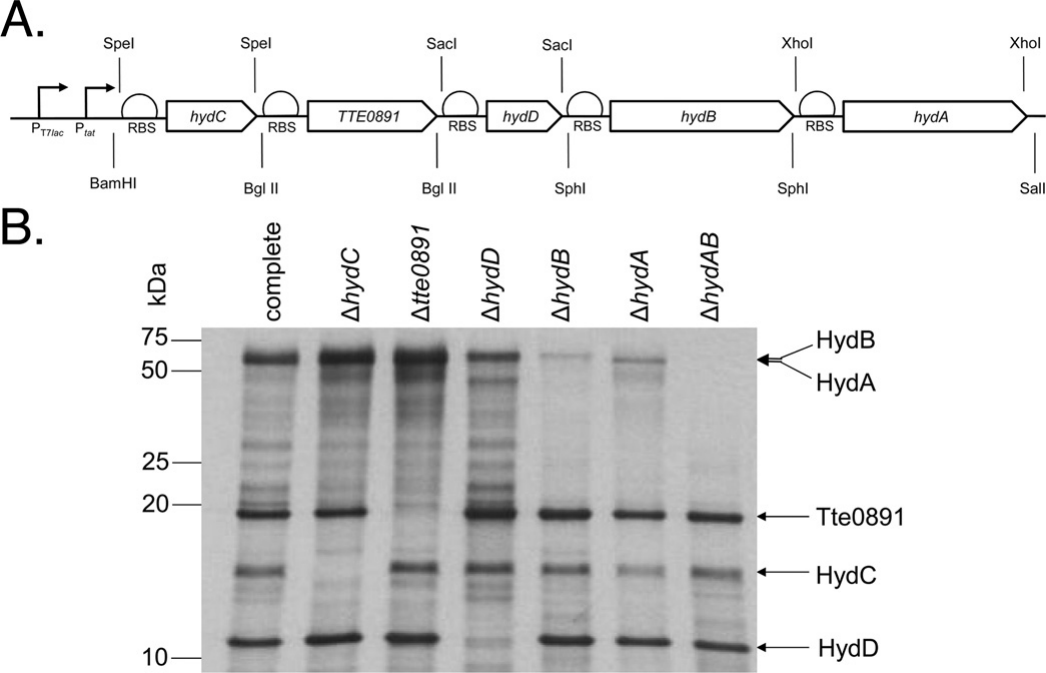
The products of a synthetic operon encoding an [FeFe]-hydrogenase are synthesised in *E. coli*. (A) The predicted structure of the synthetic operon encoding *Ca. subterranus* NADH-dependent [FeFe]-hydrogenase. Restriction sites and promoter regions are indicated. (B) The *E. coli* strain K38/pGP1-2 was transformed with plasmids: pUNI-Tte-Hyd (‘complete’); pUNI-Tte-HydΔC (‘ΔhydC’); pUNI-Tte-HydΔ0891 (‘Δ*tte0891*’); pUNI-Tte-HydΔD (‘Δ*hydD*’); pUNI-Tte-HydΔB (‘Δ*hydB*’); pUNI-Tte-HydΔA (‘Δ*hydA*’); and pUNI-Tte-HydΔAB (‘Δ*hydAB*’), grown in M9 minimal medium lacking cysteine and methionine, and labelled by the addition of ^35^S-methionine. Protein samples were then separated by SDS–PAGE (12% w/v polyacrylamide), fixed, and visualised by autoradiography.

The soluble, thermostable NADH-dependent [FeFe]-hydrogenase enzyme of *Ca. subterranus* [52] was chosen as a good candidate for a hydrogenase biased towards hydrogen production that uses a universal reductant (NADH) as a substrate. Although the *Ca. subterranus* enzyme would have a temperature optimum far above that of the normal growth conditions for *E. coli*, it was considered that any engineered enzyme could be further optimised following the initial characterisation. The constituent parts chosen to build a synthetic operon encoding this enzyme were the four proteins HydA-D as well as the hypothetical protein Tte0891, which is encoded within the native operon [52]. The primary amino acid sequences were back-translated into DNA sequence, codon optimised for *E. coli* using OPTIMIZER software [43], before a synthetic RBS and spacer sequence was included upstream of each synthetic gene: 5′-AGGAGGAAAAAAA-3′. This sequence, together with the sequence upstream of the RBS and spacer and the coding sequence itself, was then analysed using the RBS CALCULATOR software [47], which allows the efficiency of translation initiation to be predicted. The five synthetic sequences were then brought together to form a synthetic operon in which the natural gene-order was maintained (*hydC*, *tte0891*, *hydD*, *hydB*, *hydA*). Finally, unique restriction site sequences were chosen to separate each gene (Fig. 1A). The complete 5104 bp synthetic operon was then synthesised and cloned resulting in the vector pUNI-Tte-Hyd, which also contains a constitutive *tat* promoter (from *E. coli*) and a T7 promoter upstream of the synthetic genes (Table 1).

In order to validate that each gene in the synthetic operon was being correctly transcribed and translated the engineered restriction sites were used to further modify the pUNI-Tte-Hyd plasmid. A bank of six derivatives were constructed each carrying specific gene deletions in each of the five synthetic genes, as well as a Δ*hydAB* double deletion version (Table 1). The seven synthetic constructs were next used in ^35^S-methionine radiolabelling experiments. *E. coli* strain K38 (containing plasmid pGP1-2, a plasmid that encodes T7 polymerase) was transformed separately with pUNI-Tte-Hyd and the six deletion derivatives. Following pulse-labelling, SDS–PAGE and autoradiography protein products could be visualised and assigned to each gene product (Fig. 1B). In each case, the gene products migrated close to their theoretical mass by SDS–PAGE (Fig. 1B). This technique established that transcription and translation of this synthetic operon, the DNA sequence of which does not exist in nature, was possible in an *E. coli* host and results in the synthesis of apparently stable protein products.

### 3.2 Design, construction and optimization of a synthetic [FeFe] cofactor assembly operon

**Fig. 2:**
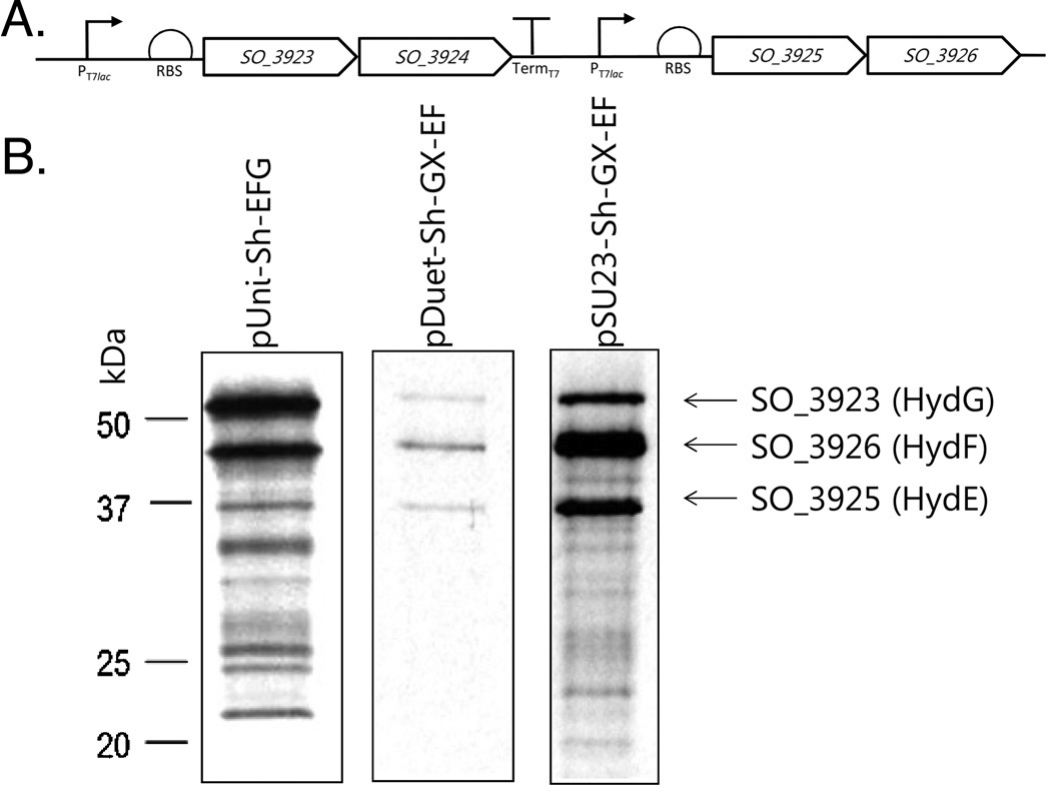
A construct for production of accessory genes required for [FeFe]-hydrogenase activity. (A) A construct encoding two bicistronic operons for *Sh. oneidensis hydGX* and *hydEF* was designed. The locations of promoters and engineered ribosome binding sites are shown. (B) The *Sh. oneidensis hydG* and *hydEF* genes are transcribed and translated*. E. coli* strain K38/pGP1-2 was transformed with plasmids: pUNI-Sh-EFG; pDuet-Sh-GX-EF; and pSU23-Sh-GX-EF and cultured in M9 minimal medium lacking cysteine and methionine and, where appropriate supplemented with 1 mM IPTG to de-repress LacI encoded on the pDuet-Sh-GX-EF plasmid. Cells were pulse-labelled with ^35^S-methionine and protein samples were then separated by SDS–PAGE (14% w/v polyacrylamide), fixed, and visualised by autoradiography.

A set of accessory proteins is required in order to assemble the special ‘H-cluster’ found in the active site of [FeFe]-hydrogenases [8]. Most biological systems utilize the activity of three accessory proteins – HydE, -F, and -G. Both HydE and HydG are radical SAM (S-adenosyl methionine) enzymes, while HydF is predicted to be a GTPase that also has a scaffolding role for the immature cofactor [8]. Recently, the *hydGXEF* genes from *Shewanella oneidensis* have been utilised in heterologous production studies of an [FeFe]-hydrogenase in *E. coli* [32]. The γ-Proteobacterium *Sh. oneidensis* is closely related to *E. coli* but, unusually for this family of prokaryotes, it encodes a periplasmic [FeFe]-hydrogenase in its genome. Initially, the putative four-gene operon containing *SO_3923* (*hydG*); *SO_3924* (*hydX*); *SO_3925* (*hydE*) and *SO_3926* (*hydF*) was amplified by PCR from *Sh. oneidensis* genomic DNA and cloned directly to give pUNI-Sh-EFG and pSU-Sh-EFG (Table 1). To examine the translational efficiency of the cloned genes, the pUNI-Sh-EFG plasmid was then used in a radiolabelling experiment in *E. coli* (Fig. 2B). In this case, not all the predicted gene products could be confidently identified using this method (Fig. 2B). To improve the expression of all the necessary genes it was decided to clone both ‘halves’ of the operon (*hydGX* and *hydEF*) separately into the dual production vector pACYCDuet-1 (Fig. 2A and Table 1), where *hydE* would contain a synthetic RBS. The pACYCDuet-1 vector has two multiple cloning sites both under the control of separate P_*T7lac*_ promoters and also encodes LacI *in cis*. Each half-operon was amplified by PCR and cloned with the initial gene in each half sharing the same RBS and spacer sequence (5′-AGGAGGAAAAAAA-3′) (Fig. 2A). The resultant plasmid, pDuet-Sh-GX-EF, was then analysed by ^35^S-Methionine radiolabelling experiments. In this case, all the three accessory proteins HydE, HydF and HydG were found to be transcribed and translated (Fig. 2B). Production of the HydX protein (23.9 kDa) was not detected (Fig. 2B).

Next, the entire P_*T7lac*_-RBS–spacer-*hydGX*- P_*T7lac*_-*hydEF* DNA fragment from pDuet-Sh-GX-EF was subcloned into pSU23 [5], which, unlike pACYC-Duet, does not encode the LacI repressor. This lead to increased production levels relative to pDuet-Sh-GX-EF as observed by radiolabelling experiments (Fig. 2B). Finally, as repression or careful induction of production of *S. oneidensis* operon was not thought to be required, the *E. coli tat* promoter (P_*tat*_) was introduced upstream of the first gene to yield pSUtat-Sh-GX-EF. This removed the need to co-produce with T7 polymerase.

### 3.3 Purification and characterization of a synthetic hydrogenase complex

**Fig. 3:**
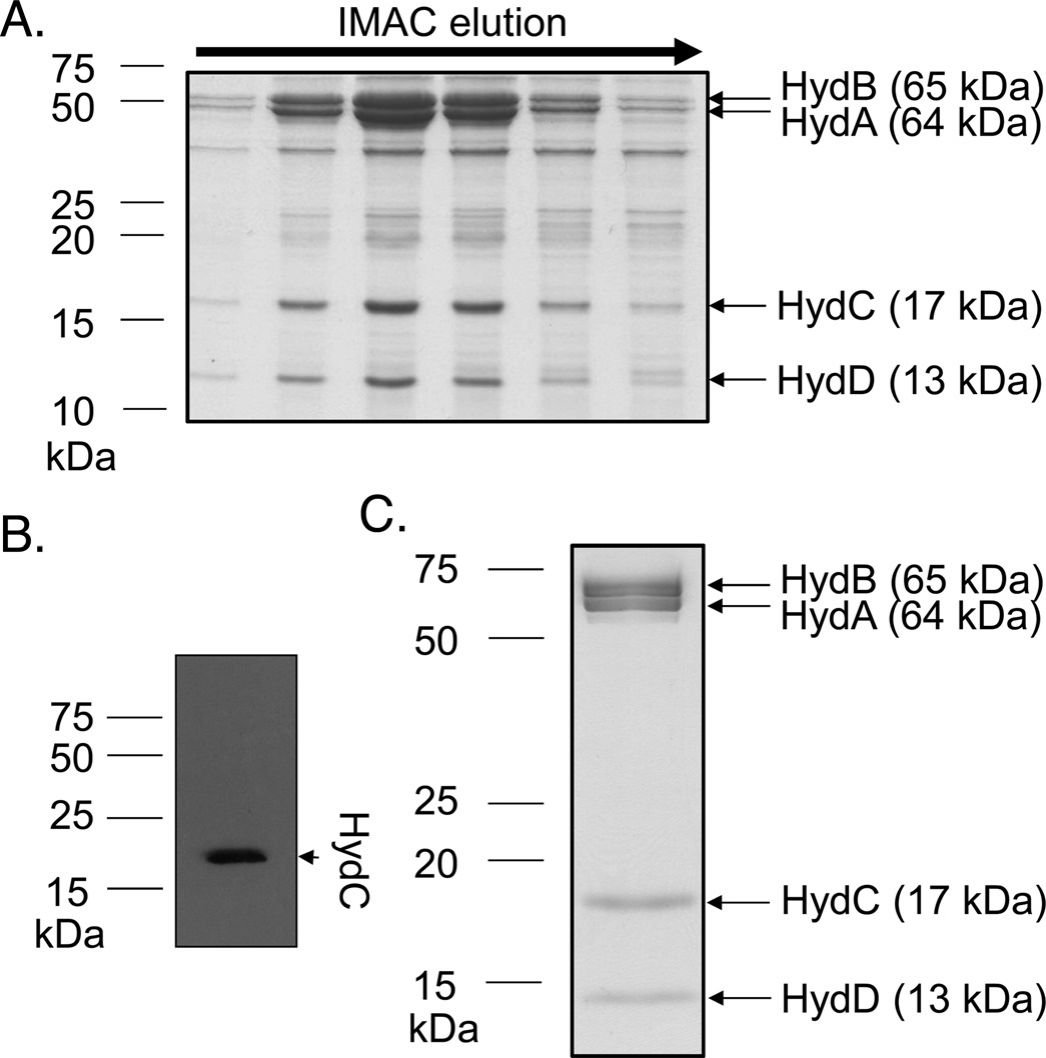
Isolation of a recombinant [FeFe]-hydrogenase. (A) *E. coli* strain PK4854 (Δ*iscR*) was transformed with plasmids: pUNI-Tte-HydhisC and pSUtat-Sh-GX-EF and cultured in LB supplemented with 0.4% (w/v) glucose, 2 mM cysteine, 2 mM ferric ammonium citrate. Cells were harvested and lysed by sonication. Crude cell extract was loaded onto a HisTrap™ HP column and eluted by an imidazole gradient and fractions (‘IMAC elution’) were collected and separated by SDS–PAGE (14% w/v acrylamide). Each subunit was identified using tryptic peptide mass fingerprinting. (B) Identification of HydC^*His*^ by Western immunoblotting. (C) SDS–PAGE analysis of the synthetic enzyme following SEC-MALLS. The peak fraction from SEC-MALLS analysis was collected, concentrated and separated by SDS-PAGE (12% w/v polyacrylamide), followed by staining with Instant Blue™.

To facilitate *in vitro* characterisation of the [FeFe]-hydrogenase, the plasmid pUNI-Tte-Hyd_hisC_ was constructed, which is identical to the pUNI-Tte-Hyd vector except that it encodes HydC^*His*^. The *E. coli* strain PK4854 (as MG1655 Δ*iscR*) was chosen as the host strain since it is de-regulated for Fe-S cluster assembly [32]. PK4854 (Δ*iscR*) was co-transformed with pSUtat-Sh-GX-EF and pUNI-Tte-Hyd_hisC_ and grown anaerobically with additional glucose, cysteine and ferric ammonium citrate. Nitrogen-saturated buffers were used during an initial immobilized metal affinity chromatography (IMAC) step. The eluted fractions exhibited a deep brown colour, and the UV/Vis absorption spectrum pointed to the presence of Fe-S clusters (Supp. Fig. S1). Analysis of the eluted peak fractions by SDS–PAGE revealed several proteins of the expected molecular masses of the NADH-dependent [FeFe]-hydrogenase (Fig. 3A). The identity of HydA, HydB and HydD was established by tryptic peptide mass-fingerprinting. The His-tagged HydC protein was located by Western immunoblot (Fig. 3B).

To assess the molecular mass of the [FeFe]-hydrogenase complex Size-Exclusion Chromatography–Multi-Angle Laser Light Scattering (SEC-MALLS) experiments were carried out on the purified enzyme. Upon elution from the SEC-MALLS column the purified enzyme appeared to form a stable large monodisperse complex containing each of the four subunits (established by SDS–PAGE; Fig. 3C) with an apparent molecular weight of 325 (±0.1%) kDa and a hydrodynamic radius of 11.9 (±3.3%) nm.

**Fig. 4:**
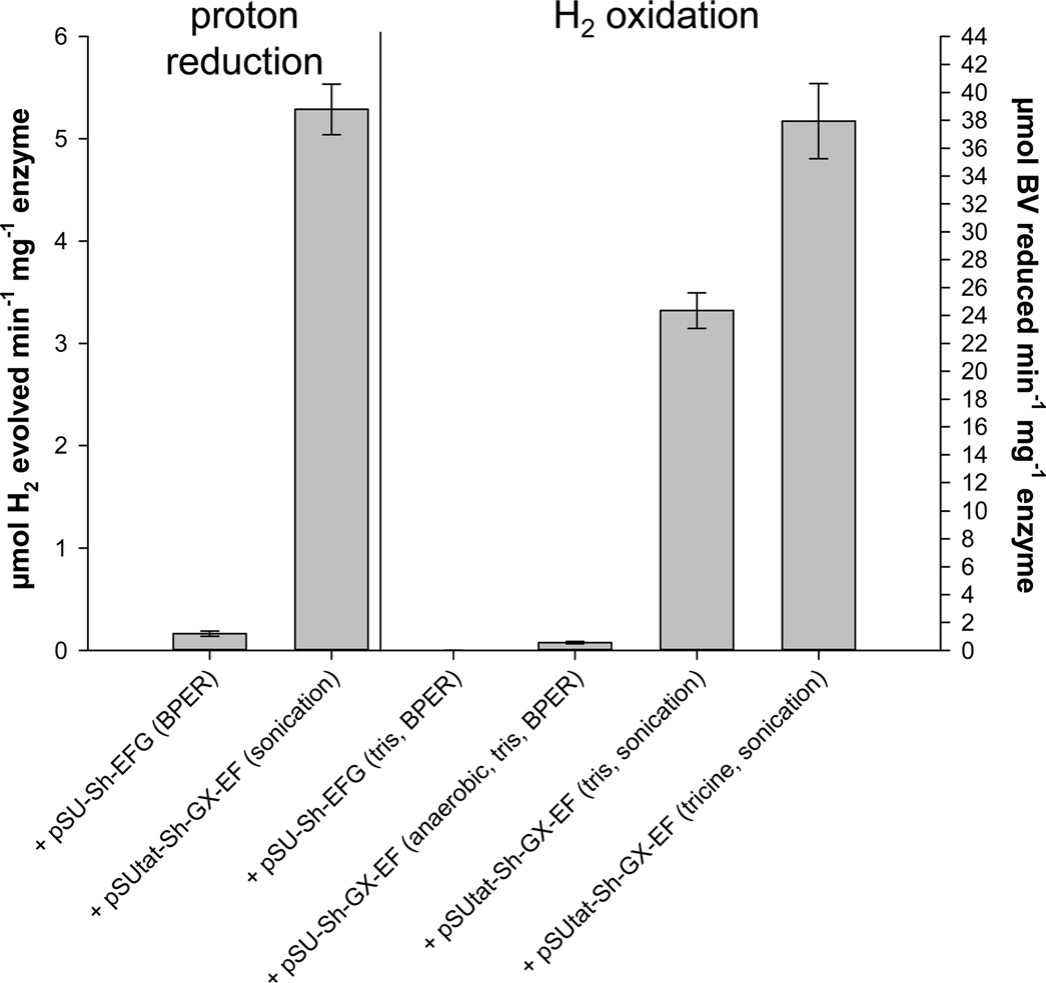
Purified synthetic [FeFe]-hydrogenase displays hydrogenase activity *in vitro*. *E. coli* strain PK4854 was co-transformed with pUNI-Tte-Hydhisc and one of three accessory plasmids: pSU-Sh-EFG; pSU23-Sh-GX-EF; or pSUtat-Sh-GX-EF, as indicated. Harvested cells were lysed either by sonication or using a chemical cocktail (BPER, Thermo Scientific) as indicated and enzyme isolated by IMAC. ‘Proton reduction’ activity involved methyl viologen-dependent H_2_ production measured in a modified Clark-type electrode. The reaction was initiated by the addition of 10 μg of purified enzyme. ‘H_2_ oxidation’ assays involved H_2_-dependent benzyl viologen reduction monitored at 578 nm in a UV-vis spectrometer. The reaction was started by the addition of 20 μg of purified enzyme and recorded at 37 °C. Error bars represent the standard error of three independent experiments.

For hydrogen oxidation assays benzyl viologen (BV) was chosen as the electron acceptor because its standard reduction potential (E_0_′ −348 mV) is more positive than that of the H^+^/½ H_2_ redox couple (E_0_′−420 mV). The His-tagged enzyme purified from cells co-producing pSUtat-Sh-GX-EF clearly catalysed the reduction of BV with H_2_ as the electron donor at 37 °C (Fig. 4). Surprisingly, enzyme prepared from cells lysed by sonication displayed BV-linked activity >40 times greater than that isolated using a detergent-based chemical cocktail (Fig. 4).

For hydrogen evolution experiments methyl viologen (MV) was chosen as the electron donor (E_0_′ = −443 mV). The His-tagged [FeFe]-hydrogenase purified using the sonication method demonstrated H_2_-evolution activity with reduced MV as the artificial electron donor (Fig. 4). Again, performing cell lysis with a chemical cocktail apparently inactivated the enzyme (Fig. 4).

### 3.4 Towards an engineered strain for Bio-H_2_ production

Having established that the [FeFe]-hydrogenase could be assembled in *E. coli*, the next step was to move away from antibiotic resistance-encoding multicopy plasmids and integrate the synthetic operon into the chromosome. The *E. coli* strain FTD147 (Δ*hyaB*, Δ*hybC*, Δ*hycE*) was chosen as a host since it contains no endogenous hydrogenase activity [44]. Using homologous recombination, the precise replacement of the *adhE* gene by the synthetic [FeFe]-hydrogenase operon was achieved and the new strain was called FTD147h3 (Table 1). In this strain the synthetic operon retains the constitutive *tat* promoter, but is also correctly positioned to be driven by the native *adhE* promoters.

**Fig. 5:**
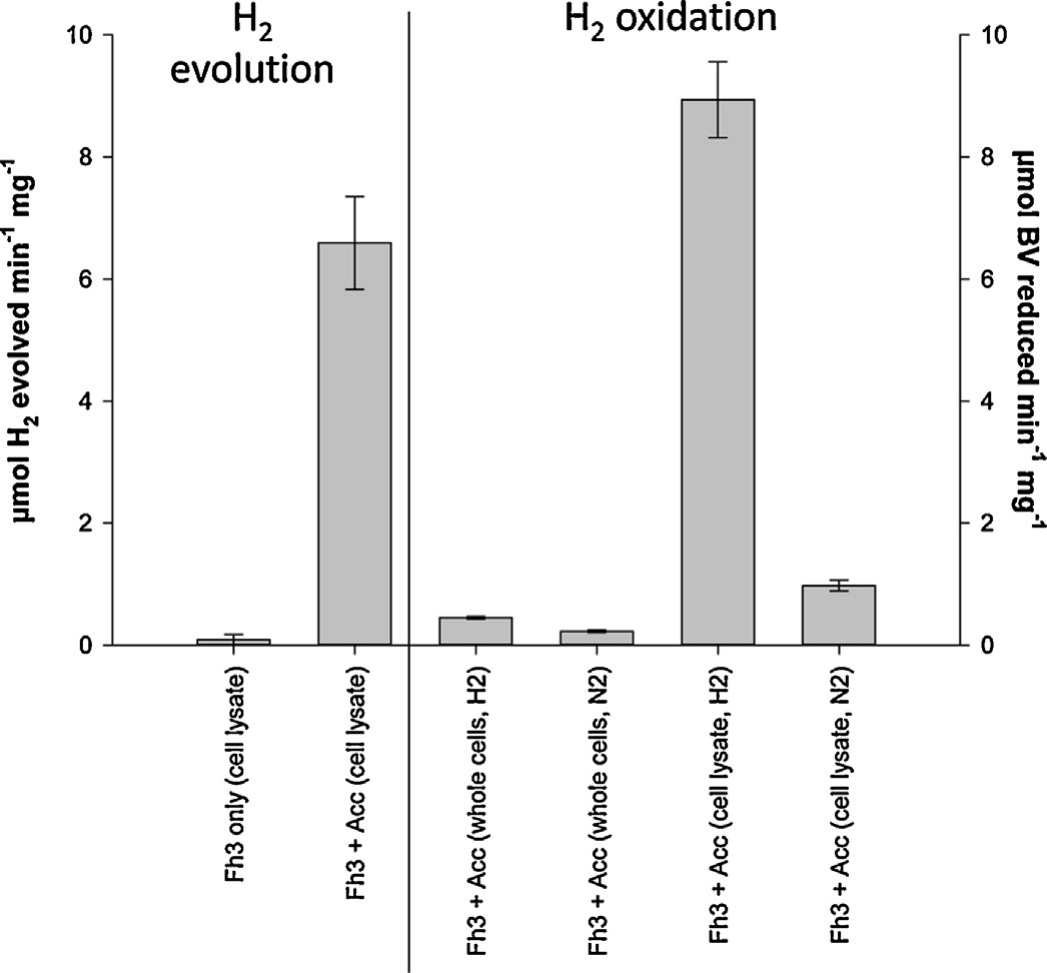
The engineered FTD147h3 strain displays hydrogenase activity. FTD147h3 (‘Fh3’) alone, or the strain transformed with pSUtat-Sh-GX-EF encoding accessory genes (‘Acc’), was cultured anaerobically in 0.4% (w/v) glucose, 2 mM cysteine, 2 mM ferric ammonium citrate. Cells were lysed by sonication resulting in a crude cell lysate, which was assayed for hydrogenase activity. ‘H_2_ evolution’ activity refers to methyl viologen-dependent H_2_ production measured in a modified Clark-type electrode. ‘H_2_ oxidation’ activity refers to H_2_-dependent benzyl viologen reduction monitored at 578 nm. N_2_-saturated buffer (‘N2’) and unbroken intact cells (‘whole cells’) were used as controls. Error bars represent the standard error of three independent experiments.

To establish whether the [FeFe]-hydrogenase was produced and active in FTD147h3 under physiological conditions for the *E. coli* host, the strain was transformed with pSUtat-Sh-GX-EF and anaerobic cultures prepared that had been supplemented with 0.4% (w/v) glucose, 2 mM cysteine and 2 mM ferric ammonium citrate. Cell pellets were flushed with argon throughout to maintain anaerobic conditions and cells were lysed by sonication. The FTD147h3 + pSUtat-Sh-GX-EF crude cell extract catalysed the reduction of BV with H_2_ as the electron donor at 37 °C (Fig. 5B). Assays were repeated in N_2_-saturated buffer to confirm H_2_-specific BV reduction (Fig. 5B). Intact whole cells were also used as negative controls (Fig. 5B), as oxidised BV is probably impermeable to the inner membrane [17]. The FTD147h3 + pSUtat-Sh-GX-EF crude cell extract also demonstrated H_2_-evolution activity with reduced MV as the artificial electron donor at 37 °C, and this activity was dependent upon co-production of the *Sh. oneidensis hydEXFG* accessory genes (Fig. 5A).

Having established that the chromosomally-encoded [FeFe]-hydrogenase is assembled and active in *E. coli* the next step was to assess Bio-H_2_ production *in vivo*. The FTD147h3 + pSUtat-Sh-GX-EF was grown anaerobically in rich medium. The culture headspace was then tested for the presence of H_2_ using gas chromatography, however no H_2_ production was observed.

### 4 Genetic evidence for an electron-confurcating mechanism for the Ca. subterranus [FeFe]-hydrogenase

Although originally reported as being able to utilise NADH as the sole electron donor for H_2_ production [52], the subunits of the *Ca. subterranus* [FeFe]-hydrogenase complex under investigation here share sequence identity with the *Th. maritima* [FeFe]-hydrogenase, which exhibits an electron-confurcating mechanism of hydrogen production involving reduced ferredoxin in combination with NADH as electron donors [51]. In the case of the *Th. maritima* system, reduced ferredoxin is a product of the oxidation of pyruvate (*E*_m_ −500 mV) catalysed by pyruvate ferredoxin oxidoreductase (POR):

pyruvate + CoA + 2Fd^ox^ ↔ acetyl-CoA + CO_2_ + H^+^ + 2Fd^red^

Therefore, given the increasing body of research highlighting bifurcating or confurcating mechanisms for cytoplasmic [FeFe]-hydrogenases [9], [27], [45], [50], it was considered possible that the *Ca. subterranus* [FeFe]-hydrogenase under experimentation here was also an electron-bifurcating/confurcating enzyme.

To address this hypothesis directly, the *Th. maritima* gene *tm0927* (encoding a ferredoxin) and the *tm0015-tm0018* operon (encoding the *γ*, *δ*, *α* and *β* subunits of a pyruvate-ferredoxin oxidoreductase) [31] were cloned both separately and together in production vectors. Successful heterologous production of the *Th. maritima* ferredoxin and the POR subunits was established using ^35^S-methionine radiolabelling (Supp. Fig. S2).

Next, the FTD147h3 strain was co-transformed with a plasmid encoding accessory genes (pSUtat-Sh-GX-EF) and a plasmid encoding *Th. maritima* POR and ferredoxin (pUNI-Tm-POR-Fd). The strain was grown anaerobically in a supplemented minimal medium containing casamino acids and 0.8% (w/v) glucose. A minimal medium was chosen to allow further analysis of metabolites in the growth medium by HPLC, however supplementation of the medium with casamino acids was required to obtain some growth in the absence of *adhE*. Following incubation for 17 h the culture headspace was assayed for the presence of H_2_ by gas chromatography (Table 2). Compared to the control strains that do not produce H_2_, co-production of POR and Fd with the activated [FeFe]-hydrogenase in FTD147h3 increased *in vivo* H_2_ production to detectable levels, reaching 7.4 ± 4.1 nmols H_2_ OD_600_^−1^ ml^−1^ (Table 2). Please note that this is a single-end-point assay that determines the total amount of H_2_ in the culture headspace following growth. The values are normalised for cell growth and culture size to allow for comparison between different strains, however it is not possible to determine biochemical reaction rates from such a single-point assay.

In an attempt to increase this amount of H_2_ production a mutation in *pflA* was incorporated into the FTD147h3 strain to give CPD159F (Table 1). PflA is the pyruvate formatelyase (PflB) activating enzyme, which is required for anaerobic metabolism of pyruvate to generate formate and acetyl CoA. Here, the hypothesis was that prevention of disproportionation of pyruvate by the natural route should force more pyruvate towards POR and the synthetic hydrogenase system. Unfortunately, H_2_ production was not further boosted by adding a *pflA* mutation (Table 2), instead the mutation has the effect of inducing lactate production in the growth medium as assayed by HPLC, presumably by induction or activation of lactate dehydrogenases (Table 2).

Next, a version of FTD147h3 was prepared that was devoid of the *iscR* gene. IscR is the key regulator (repressor) of genes required for Fe-S cluster biosynthesis and some other metalloenzymes [21]. The genetic inactivation of *iscR* de-regulates the Fe-S cluster assembly machinery and, as a result, the new CLK001 strain (Table 1) would be expected to be more efficient at Fe-S protein biosynthesis [32]*.* The CLK001 strain was co-transformed with a plasmid encoding accessory genes (pSUtat-Sh-GX-EF) and a plasmid encoding *Th. maritima* POR and ferredoxin (pUNI-Tm-POR-Fd) and the culture headspace assayed for H_2_ gas following growth in casamino acid-supplemented minimal medium (Table 2). Again, compared to control strains, co-production of POR and Fd in CLK001 increased the amount of H_2_ produced *in vivo* to detectable levels, reaching 9.0 ± 4.0 nmols H_2_ OD_600_^−1^ ml^−1^ (Table 2), which is not significantly different to the H_2_ levels recorded for the FTD147h3 strain (Table 2). Further modification of CLK001 by incorporation on a *pflA* mutation to yield strain CPD152E (Table 1) did not increase the final amount of H_2_ produced (Table 2) but again led to an increase in lactate found secreted into the growth medium (Table 2).

Finally, the four engineered strains were transformed with the accessory plasmid and the plasmid encoding POR and ferredoxin before being cultured anaerobically in the rich medium TGYEP (Table 3). Growth in such rich medium means that metabolite quantification of the spent media by HPLC is not practical, however headspace analysis by gas chromatography was still possible (Table 3). In this case, all of the engineered strains ceased H_2_ production except those carrying an *iscR* modification (Table 3). Here, co-production of POR and Fd in CLK001 resulted in the amount of H_2_ produced *in vivo* elevated to 28.7 ± 5.1 nmols H_2_ OD_600_^−1^ ml^−1^ (Table 3).

## 5 Discussion

### 5.1 Characterization of a synthetic [FeFe]-hydrogenase.

In this work, a synthetic approach to molecular genetics has been partnered with a more traditional biochemical characterization of the products of those synthetic genes. A synthetic operon was designed, which does not exist in nature, and shown to encode all expected polypeptides in radiolabelling experiments. In turn, those polypeptides assemble into an active [FeFe]-hydrogenase when co-expressed with accessory genes from *Sh. oneidensis*. In the course of this work, attempts were made to utilise homologs of the *hydEFG* genes from *Ca. subterranus* instead. This was complicated by the fact that the *Ca. subterranus* genome does not contain an obvious *hydEFG* operon. Instead four separate genes were identified including *tte1885* (encoding a protein most like HydE from *C. acetylicum*); *tte0814* (encoding a GTPase most like HydF from *C. acetylicum*); *tte1568* (most like HydG from *C. acetylicum*); and *tte1073* (most like the structurally-defined HydE from *Th. maritima*, but also related to HydG from *C. actylicum* and Tte1885). A synthetic operon encoding these gene products was designed and assembled, however none of the polypeptides were expressed and so no [FeFe]-hydrogenase activation was observed.

Using the *Sh. Oneidensis* biosynthetic machinery allowed the isolation of enzyme that had hydrogen oxidation and proton reduction activities with redox dyes, and with a mass of 325 kDa the enzyme was demonstrated to adopt a (HydABCD)_2_ conformation. Moreover, the synthetic operon encoding the enzyme could be stably integrated into the *E. coli* chromosome, thus taking the first step towards generating a synthetic strain freed from mobile genetic elements carrying antibiotic resistance.

### 5.2 Re-wiring *E. coli* metabolism for H_2_ production.

Engineering bacterial hydrogen production has been a long-standing aim for some microbial biotechnologists. Common approaches have been to direct natural electron and carbon flux towards the native hydrogenases of *E. coli* by genetic and metabolic engineering [37], [40], [55]. In addition, overproduction of heterologous hydrogenases in *E. coli* has also been attempted with various degrees of success. For example, simple production of the genes encoding the large and small subunits of the Tat-dependent, O_2_-tolerant, respiratory [NiFe]-hydrogenase from *Hydrogenovibrio marinus* was sufficient to increase H_2_ production [29], even though the source of reductant was not clear and the metallocofactor-deficient BL21 strain was used as a chassis [41].

The deliberate connection of a hydrogenase directly to a source of electrons, and in particular a source of reductant involved in central energy metabolism, is an attractive proposition. In this work, the NADH pool was chosen since recycling on NAD^+^ is critical for fermentative growth and NADH biochemistry is linked directly to H_2_ metabolism in many microbial systems. The cytoplasmic concentration of NADH cofactor is 1.4 nmoles/mg dry weight of *E. coli* grown under anaerobic fermentative conditions, and NAD^+^ is 4.8 nmoles/mg dry weight [34], [57]. Under anaerobic fermentative conditions this ratio is maintained primarily by the action of AdhE in *E. coli* [57]. In the absence of active AdhE, the synthetic [FeFe]-hydrogenase designed in this work was unable to generate hydrogen. This was initially surprising since in an earlier study the isolated native enzyme was able to produce H_2_ *in vitro* with NADH as electron donor [52]. Moreover, the soluble NADH-dependent [NiFe]-hydrogenase from *Ralstonia eutropha* was, following engineering, found to be activatable in *E. coli* and was successful in generating increased H_2_ in an *adhE* mutant [20]. In comparison, these data suggest that the synthetic [FeFe]-hydrogenase under investigation here was not operating at all efficiently as a stand-alone NADH-linked hydrogenase, and may be lacking in some vital components for its activity *in vivo*.

### 5.3 Heterologous production of a putative electron-confurcating hydrogenase for engineering H_2_ production

In the original *Ca. subterranus* hydrogenase characterisation [52], 1 mM Ti(III) citrate was added during the NADH-dependent H_2_ production assays [52]. Ti(III) citrate is a powerful reducing agent (E_0_′ = -500 mV), and this perhaps gives an initial hint that an additional source of electrons was needed for this enzyme to operate correctly. However, it should be noted that the enzyme was capable of the reverse reaction, H_2_-dependent reduction of NAD(P)^+^, without a second electron acceptor present [52], although this reaction would be thermodynamically favourable under the conditions used. Moreover, Schut and Adams [51] characterised the NADH-linked [FeFe]-hydrogenase from *Th. maritima*, which is an enzyme closely related to the *Ca. subterranus* hydrogenase at the amino acid level. In *Th. maritima* the [FeFe]-hydrogenase accepts a second source of electrons, this time from a high potential reduced ferredoxin (*E*_m _= −453 mV) [51], that acts as a thermodynamic driver thus allowing oxidation of NADH linked to H_2_ production [51].

**Fig. 6:**
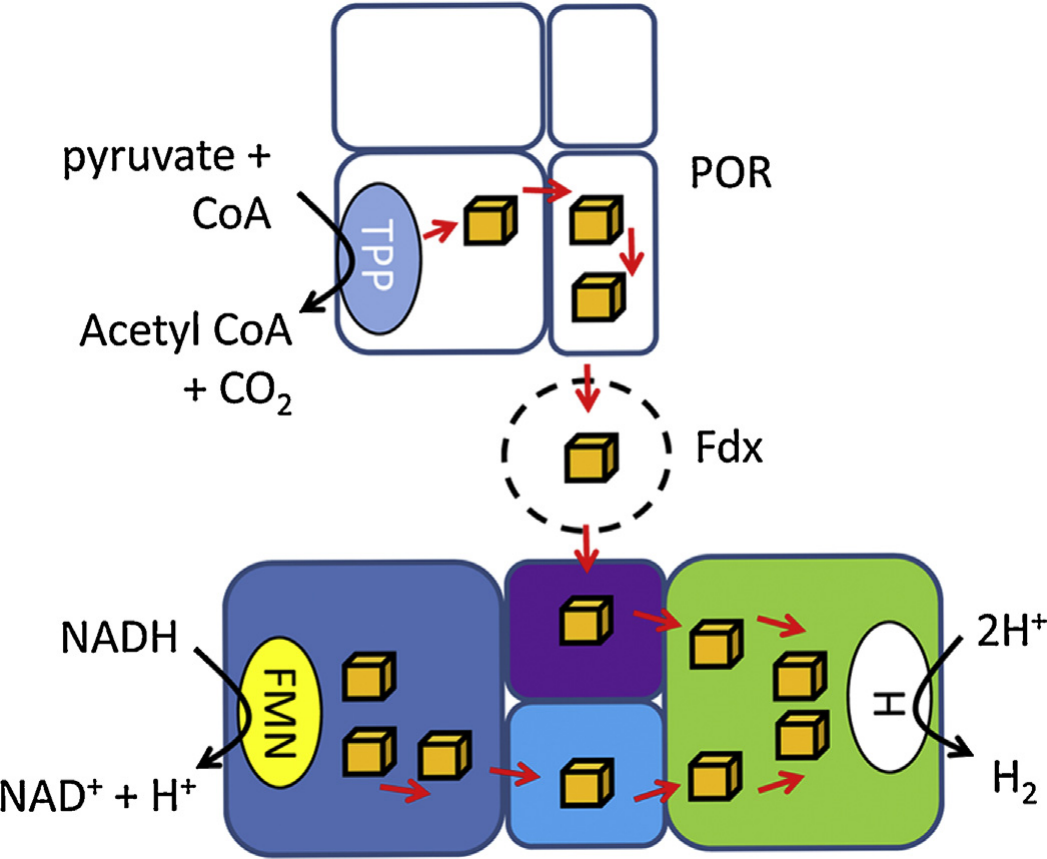
Hydrogen production by electron confurcation. Hypothetical model depicting the H_2_ production system engineered into *E. coli* based on collated evidence from several biological systems. A blue oval depicts the presence of a thiamine pyrophosphate (TPP) cofactor, the fellow oval represents flavin mononucleotide (FMN) and the white oval the ‘H’-cluster. Iron-sulfur clusters are represented by brown cubes. The hypothetical direction of electron flow is suggested by red arrows.

The possibility that the synthetic *Ca. subterranus* enzyme studied here also required a second input of electrons was tested directly here using a genetics-based *in vivo* approach. The genes encoding *Th. maritima* POR and ferredoxin were cloned onto a single vector and co-produced with the synthetic *Ca. subterranus* enzyme in *E. coli*. The engineered strain generated a small amount of H_2_ that could be increased by the addition of an *iscR* mutation (Table 2, Table 3). The amounts of hydrogen produced are small compared to what native *E. coli* can produce under similar growth conditions (Table 2, Table 3). For example, in rich media the essentially wild-type control strain produced around 5 μM H_2_ per OD unit per ml of culture (Table 3), which is around 180 times more than the engineered system. These results should be considered as proof-of-concept, highlighting that complex electron bifurcating systems can be engineered, but given the low amounts of H_2_ produced it is clear that the enzymes and strains involved will need further optimization to provide increased levels of Bio-H_2_. What these data clearly show is that the synthetic *Ca. subterranus* enzyme is most likely an electron confurcating/bifurcating system *in vivo* (Fig. 6), and this adds fresh backing to the original hypothesis on the physiological role of this enzyme in its native system [51]. While pyruvate oxidation has been linked directly to H_2_ production in a metallocofactor-deficient *E. coli* host previously [3], [41], this is, to our knowledge, the first synthetic engineering of an active electron confurcating/bifurcating [FeFe]-hydrogenase in a heterologous host. This system also allows a genetic system for the characterisation of such complex [FeFe]-hydrogenases, which may be of use to many other biological systems. The ability to connect additional H_2_-producing capabilities directly to central energy, carbon and cofactor metabolism may have the potential to be harnessed in future bioenergy research projects.

## Figures and Tables

**Table 1:** Strains and plasmids utilized in this work.

Strain	Relevant genotype	Source
MC4100	*E. coli* K-12. F^−^, *λ*^−^, [*araD139*]_B/r_, Δ(*argF-lac*)U169, *e14*-, *flhD5301*, Δ(*fruK*-*yeiR*) 725(*fruA25*), *relA1*, *rpsL150*(Str^R^), *rbsR22*, Δ(*fimB*-*fimE*)*632*(*::IS1*), *deoC1*	[11]
K38	*E. coli* K-12. HfrC, *phoA4*, *pit-10*, *tonA22*, *ompF627*, *relA1*	[36]
FTD147	as MC4100, Δ*hyaB*, Δ*hybC*, Δ*hycE*	[16]
Teatotal1	as FTD147, Δ*adhE*	this work
FTD147h3	as FTD147, Δ*adhE::*(*hydC-tte0891-hydD-hydB-hydA*)	this work
CPD159F	as FTD147h3, *pflA::*Tn5	this work
CLK001	as FTD147h3, Δ*iscR*	this work
CPD152E	as CLK001, Δ*pflA::*Kan	this work
PK4854	*E. coli* K-12. F^−^, λ^−^, *ilvG*^−^, *rfb-50*, *rph-1*, Δ*iscR*	[21]

Plasmid	Relevant genotype	Source
pGP1-2	encoding T7 polymerase (Kan^R^)	[53]
pUNI-PROM	as pT7.5 (Amp^R^, P_T7_, P_*tat*_)	[28]
pUNI-Tte-Hyd	as pUNI-PROM, synthetic operon encoding *Ca. subterranus* HydC, Tte0891, HydD, HydB, HydA	this work
pUNI-Tte-HydΔA	as pUNI-Tte-Hyd, Δ*hydA*	this work
pUNI-Tte-HydΔB	as pUNI-Tte-Hyd, Δ*hydB*	this work
pUNI-Tte-HydΔAB	as pUNI-Tte-Hyd, Δ*hydAB*	this work
pUNI-Tte-HydΔC	as pUNI-Tte-Hyd, Δ*hydC*	this work
pUNI-Tte-HydΔD	as pUNI-Tte-Hyd, Δ*hydD*	this work
pUNI-Tte-HydΔ0891	as pUNI-Tte-Hyd, Δ*tte0891*	this work
pUNI-Tte-Hyd_hisC_	as pUNI-Tte-Hyd, with hexa-His affinity tag sequence in *hydC*	this work
pUNI-Sh-EFG	as pUNI-PROM, natural *Sh. oneidensis* SO_3923 (*hydG*); SO_3924 (*hydX*); SO_3925 (*hydE*); and SO_3926 (*hydF*) operon	this work
pUNI-Tm-POR-Fd	as pUNI-PROM, natural *Th. maritima tm0015*-*tm0018* operon (encoding POR), and *tm0927* (ferredoxin)	this work
pSU-PROM	as pSU40 (Kan^R^, P_*tat*_)	[28]
pSU-Sh-EFG	as pSU-PROM with insert fragment from pUNI-Sh-EFG	this work
pACYCDuet-1	as pACYC184 (Cm^R^, P_T7*lac*-1_, P_T7*lac*-2_, *lacI*^+^)	Novagen
pDuet-Sh-GX-EF	as pACTC-Duet-1, with natural *Sh. oneidensis hydGX* and *hydEF* cloned under separate T7 promoters	this work
pSU23	Cm^R^, P_T7*lac*_	[5]
pSU23-Sh-GX-EF	as pSU23 with insert fragment from pDuet-SH-GX-EF	this work
pSUtat-Sh-GX-EF	As pSU23-Sh-GX-EF with *E. coli tat* promoter	this work

**Table 2:** Hydrogen production and metabolite analysis after anaerobic growth in supplemented M9 media.

strain^a^	Relevant genotype	hydrogen^b^	pyruvate^c^	succinate^c^	lactate^c^	formate^c^	acetate^c^	OD_600nm_
		nmol OD_600_ _nm_^−1^ ml culture^−1^	mM OD_600_^−1^					
MC4100	–	8232 ± 620	0.62 ± 0.02	3.9 ± 0.2	5.9 ± 0.2	10 ± 1.3	15 ± 0.4	1.25
FTD147	Δ*hyaB*, Δ*hybC*, Δ*hycE*	<1	0.41 ± 0.04	2.2 ± 0.1	6.9 ± 0.4	21 ± 2.2	13 ± 0.3	1.30
Teatotal1	Δ*hyaB*, Δ*hybC*, Δ*hycE*, Δ*adhE*	<1	0.05 ± 0.01	2.5 ± 0.1	2.9 ± 0.0	3.8 ± 0.1	12 ± 0.5	0.49
FTD147h3	Δ*hyaB*, Δ*hybC*, Δ*hycE*, Δ*adhE*::*hydABCD*	<1	0.03 ± 0.03	2.9 ± 0.5	3.7 ± 0.8	3.6 ± 0.6	11 ± 0.5	0.47
FTD147h3 + p^d^	Δ*hyaB*, Δ*hybC*, Δ*hycE*, Δ*adhE*::*hydABCD*	7.4 ± 4.1	0.2 ± 0.17	3.2 ± 0.9	5.8 ± 1.4	6.1 ± 2.6	8.2 ± 0.4	0.34
CPD159F	Δ*hyaB*, Δ*hybC*, Δ*hycE*, Δ*adhE*::*hydABCD*, *pflA*	<1	0.68 ± 0.03	2.4 ± 0.1	26 ± 0.1	1.2 ± 0.3	4 ± 0.1	1.03
CPD159F + p^d^	Δ*hyaB*, Δ*hybC*, Δ*hycE*, Δ*adhE*::*hydABCD*, *pflA*	5.5 ± 1.5	0.73 ± 0.03	2.2 ± 0.2	24 ± 3	3.3 ± 0.5	3.3 ± 0.0	0.81
CLK001	Δ*hyaB*, Δ*hybC*, Δ*hycE*, Δ*adhE*::*hydABCD*, Δ*iscR*	<1	0.07 ± 0.05	2.9 ± 0.3	4.7 ± 0.3	3.3 ± 0.4	9.5 ± 0.4	0.45
CLK001 + p^d^	Δ*hyaB*, Δ*hybC*, Δ*hycE*, Δ*adhE*::*hydABCD*, Δ*iscR*	9.0 ± 4.0	0.06 ± 0.03	2.7 ± 0.2	5.3 ± 1.2	4 ± 0.4	7.8 ± 0.1	0.49
CPD152E	Δ*hyaB*, Δ*hybC*, Δ*hycE*, Δ*adhE*::*hydABCD*, Δ*iscR*, Δ*pflA*	<1	1 ± 0.17	3 ± 0	27 ± 0.8	1.3 ± 0.2	4.4 ± 0.3	0.97
CPD152E + p^d^	Δ*hyaB*, Δ*hybC*, Δ*hycE*, Δ*adhE*::*hydABCD*, Δ*iscR*, Δ*pflA*	7.9 ± 0.2	n.d.	n.d.	n.d.	n.d.	n.d.	0.77

The mean and standard deviation of at least three independent measurements are shown.

n.d.—not determined.

a Cultures were grown anaerobically in supplemented M9 in closed Hungate tubes at 37 °C for 17 h.

b H_2_ content was determined from headspace gas phase by gas chromatography.

c Metabolites were determined from cell-free culture supernatants by HPLC.

d ‘+p’ denotes addition of accessory genes (pSUtat-Sh-GX-EF) and a plasmid encoding *Th. maritima* POR and ferredoxin (pUNI-Tm-POR-Fd).

**Table 3:** Hydrogen production after anaerobic growth in rich media

strain^a^	genotype	hydrogen^b^	OD_600nm_
		nmol OD_600_^−1^ ml^−1^	
MC4100	–	5394 ± 190	1.71
FTD147	Δ*hyaB*, Δ*hybC*, Δ*hycE*	<1	1.87
Teatotal1	Δ*hyaB*, Δ*hybC*, Δ*hycE*, Δ*adhE*	<1	1.00
FTD147h3	Δ*hyaB*, Δ*hybC*, Δ*hycE*, Δ*adhE*::*hydABCD*	<1	0.40
FTD147h3 + plasmids^c^	Δ*hyaB*, Δ*hybC*, Δ*hycE*, Δ*adhE*::*hydABCD*	<1	0.59
CPD159F	Δ*hyaB*, Δ*hybC*, Δ*hycE*, Δ*adhE*::*hydABCD*, *pflA*	<1	1.70
CPD159F + plasmids^c^	Δ*hyaB*, Δ*hybC*, Δ*hycE*, Δ*adhE*::*hydABCD*, *pflA*	<1	1.53
CLK001	Δ*hyaB*, Δ*hybC*, Δ*hycE*, Δ*adhE*::*hydABCD*, Δ*iscR*	<1	0.63
CLK001 + plasmids^c^	Δ*hyaB*, Δ*hybC*, Δ*hycE*, Δ*adhE*::*hydABCD*, Δ*iscR*	28.7 ± 5.1	0.48
CPD152E	Δ*hyaB*, Δ*hybC*, Δ*hycE*, Δ*adhE*::*hydABCD*, Δ*iscR*, Δ*pflA*	<1	1.59
CPD152E + plasmids^c^	Δ*hyaB*, Δ*hybC*, Δ*hycE*, Δ*adhE*::*hydABCD*, Δ*iscR*, Δ*pflA*	8.4 ± 3.3	1.35

The mean and standard deviation of at least three independent measurements are shown.

a Cultures were grown anaerobically in TGYEP, pH 6.5 in closed Hungate tubes at 37 °C for 17 h.

b H_2_ content was determined from headspace gas phase by gas chromatography.

c denotes addition of accessory genes (pSUtat-Sh-GX-EF) and a plasmid encoding *Th. maritima* POR and ferredoxin (pUNI-Tm-POR-Fd).
