# Supplementary material for: Integration of an [FeFe]-hydrogenase into the anaerobic metabolism of Escherichia coli
Source: Biotechnol Rep (Amst). 2015 Oct 19;8:94–104. doi: 10.1016/j.btre.2015.10.002 (PMC4694547; doi:10.1016/j.btre.2015.10.002)
Supplement: Supplementary file 1 [file mmc1.docx]

**Integration of an [FeFe]-hydrogenase into the anaerobic metabolism of *Escherichia coli***

#### Ciarán L. Kelly, Constanze Pinske, Bonnie J. Murphy, Alison Parkin, Fraser A. Armstrong, Tracy Palmer and Frank Sargent

## SUPPLEMENTARY Information


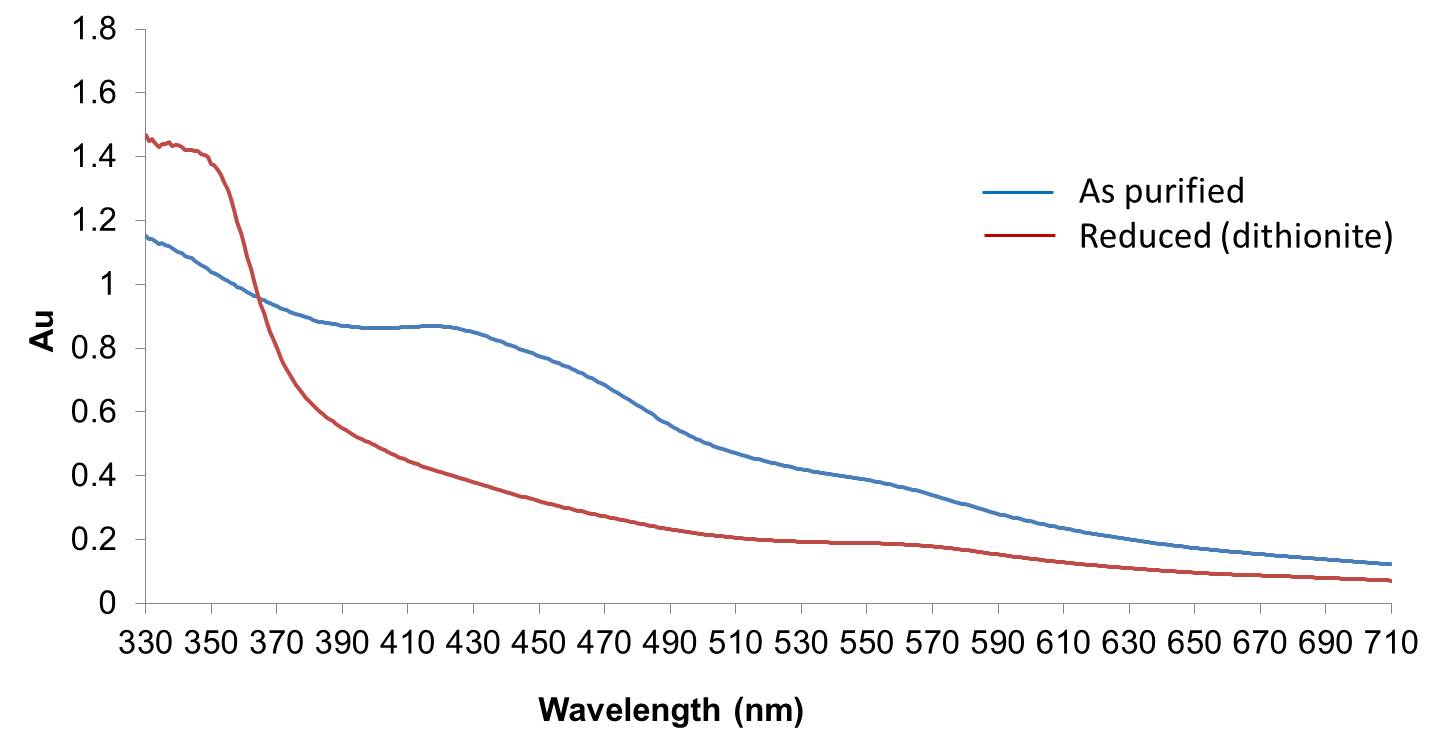


Supp. Figure S1. UV-Vis absorption spectroscopy of purified enzyme shows characteristic FeS-cluster shoulders at 420 and 450 nm. Purified enzyme was diluted 1:1 with 100 mM sodium phosphate buffer pH 6.0 and analysed over wavelengths from 330 to 710 nm.


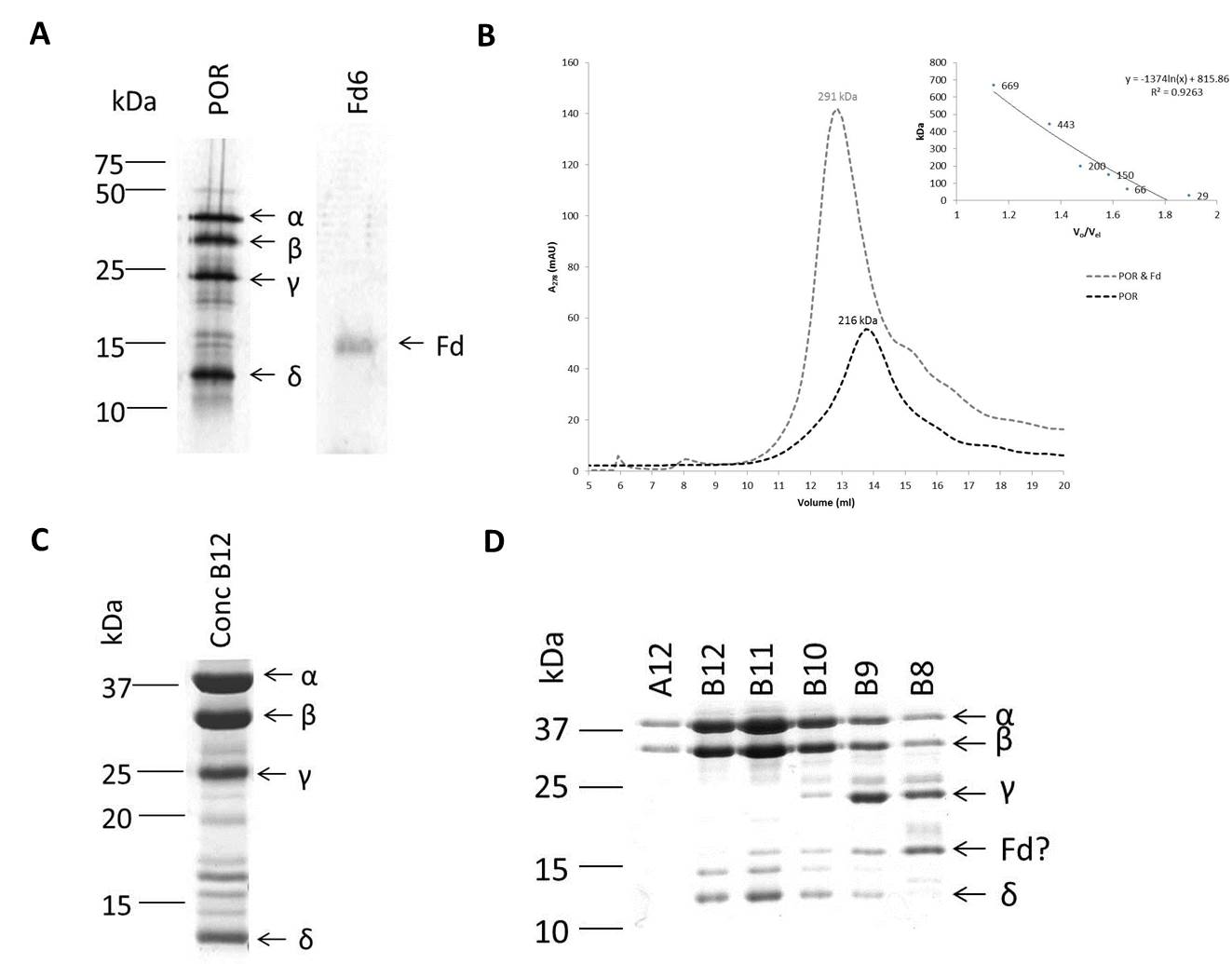


Supp Figure S2. Heterologous expression of *Th. maritima* pyruvate:ferredoxin oxidoreductase (POR) and ferredoxin (Fd). In vivo ^35^S Methionine radiolabelling of proteins encoded by plasmids pUni-Tm-POR and pUNI-Tm-Fd6 was perfomed followed by SDS-PAGE and autoradiography.
